# Combating Social Isolation among Older Immigrant Adults: A Qualitative Interpretive Meta-Synthesis

**DOI:** 10.1093/geroni/igab046.3468

**Published:** 2021-12-17

**Authors:** Vivian Miller, Betty Tonui, Dolapo Adeniji

**Affiliations:** 1 Bowling Green State University, Bowling Green, Ohio, United States; 2 Oakland University, Oakland University Rochester, Michigan, United States; 3 IUPUI, Avon, Indiana, United States

## Abstract

Older immigrants totaled 7.3 million in 2018, representing 13.9 percent of the population of seniors in the U.S. While this population is found to contribute significantly to society, along with new opportunities comes circumstantial challenges. Of these, one of the most salient issues for foreign-born older adults is social isolation. Additionally, this population may be at an increased risk for social isolation with poor mental health because migrating to a new country might results in resettlement challenges. Despite these concerns, less is known about the consequences of social isolation among older immigrant adults. Guided by the Population Interest Context (PICO) framework and the Qualitative Interpretive Meta-Synthesis (QIMS) guidelines, this study seeks to explore consequences of social isolation among older immigrant, as well as interventions to combat isolation. The final sample of six full text articles were published between 2011 and 2021, totaling 180 participants with ages ranging from 61 to 93 years old. Findings from the study indicated that older immigrants are at risk of social isolation and loneliness because they have fewer social connections due to leaving behind their familiar social group in the home country, encounter linguistic challenges that negatively contribute to greater social isolation and poor mental health. Despite these difficulties older immigrants reported various social interventions, i.e., access to senior centers, community programs and services to be of greater importance in building social networks. Authors discuss opportunities for future research, such as exploring evidence-based studies on interventions for social isolation and loneliness of older immigrant populations.

